# Morbidity and mortality in a prospective cohort of people who were homeless during the COVID-19 pandemic

**DOI:** 10.3389/fpubh.2023.1233020

**Published:** 2023-09-14

**Authors:** Sandrine Loubiere, Ikrame Hafrad, Elisabetta Monfardini, Marine Mosnier, Thomas Bosetti, Pascal Auquier, Emilie Mosnier, Aurélie Tinland

**Affiliations:** ^1^Department of Clinical Research and Innovation, Support Unit for Clinical Research and Economic Evaluation, Assistance Publique – Hôpitaux de Marseille, Marseille, France; ^2^CEReSS – Health Service Research and Quality of Life Center, EA 3279: CEReSS – Health Service Research and Quality of Life Center, School of medicine – La Timone Medical Campus, Aix-Marseille University, Marseille, France; ^3^Department of Psychiatry, Assistance Publique – Hôpitaux de Marseille, Marseille, France; ^4^Médecins du Monde – Doctors of the World, Marseille, France; ^5^INSERM, IRD, SESSTIM, Sciences Economiques & Sociales de la Santé & Traitement de l’Information Médicale, ISSPAM, Aix Marseille University, Marseille, France

**Keywords:** COVID-19, seroprevalence, morbidity, mortality, hospitalization, homelessness

## Abstract

**Introduction:**

Certain living conditions, such as homelessness, increase health risks in epidemic situations. We conducted a prospective observational cohort study to investigate the impact of the COVID-19 pandemic on morbidity and mortality in adult people who were homeless.

**Methods:**

The study population comprised around 40% of the entire population experiencing homelessness in Marseille. They were enrolled at 48 different locations during the first pandemic wave (June to August 2020) and were followed up 3 and 6 months later. Rapid serological screening for SARS-CoV-2 was performed by community outreach teams at each follow-up, who also conducted interviews. Death registers and hospital administrative databases were consulted.

**Results:**

A total of 1,332 participants [mean age 40.1 years [SD 14.2], women 339 (29.9%)] were enrolled in the cohort. Of these, 192 (14.4%) participants were found positive for COVID-19 and were propensity score matched (1:3) and compared with 553 non-COVID-19 cases. Living in emergency shelters was associated with COVID-19 infection. While 56.3% of the COVID-19-infected cohort reported no symptoms, 25.0% were hospitalized due to the severity of the disease. Presence of three or more pre-existing comorbidities was associated with all-cause hospitalization. Among COVID-19 cases, only older age was associated with COVID-19 hospitalization. Three deaths occurred in the cohort, two of which were among the COVID-19 cases.

**Conclusion:**

The study provides new evidence that the population experiencing homelessness faces higher risks of infection and hospitalization due to COVID-19 than the general population. Despite the efforts of public authorities, the health inequities experienced by people who are homeless remained major. More intensive and appropriate integrated care and earlier re-housing are needed.

## 1. Introduction

At the end of 2019, the SARS-CoV-2 virus emerged in China causing more than 6 million deaths worldwide including 145,150 in France, then the WHO declared the end of the pandemic in sight in September 2022 (1). Given the rapid increase in the number of cases, a first general lockdown was promptly implemented by the French government at the beginning of the pandemic over nearly two months, from March 17, 2020 to May 3, 2020. Two additional lockdowns took place in France from October 30, 2020 to December 15, 2020, and from April 3 to May 3, 2021. These containment measures were accompanied by a sheltering program for the homeless, with the assignment of additional emergency places, shelters and requisitioned hostels in most cities (2). These measures carried out by the public authorities were also relayed and supported by the European Federation of National Organisations Working With the Homeless (FEANTSA), as of March 2020, and by well-established associations in France, and even by health professionals and other experts in Europe (3–5).

Indeed, people who are homeless have faced permanent constraints with regard to compliance with COVID-19 restrictions: measures recommended for the general population are not easy to understand, due to structural exclusion from access to information, or even enforceable, such as handwashing, in the absence of a water point. For people experiencing homelessness, confinement was made impossible in most cases due to the absence of accessible individual accommodation solutions (6, 7). The fragmentation and remoteness of standard support structures, as well as the lack of dedicated regular places to stay, resulted in frequent mobility and in regular contact between individuals experiencing homelessness and community support service teams (during food distribution or within mobile health structures). This may have facilitated the spread of the virus (8). In addition to these environmental risk factors, people who are homeless are known to have a high prevalence of chronic diseases, particularly lung disease, addictions, and are predominantly male and aging, which are known risk factors for severe forms of COVID-19 (9–11).

A significant number of publications have focused on the relationship between homelessness and COVID-19. However, these publications have primarily studied the prevalence or seroprevalence of SARS-CoV-2 in this population. In particular, previous studies showed that the spread of COVID-19 was greater in the population experiencing homelessness than in the general population, with seroprevalence rates ranging from 4 to 36%, depending on where they were living at the time of the screening/diagnosis (i.e., rates at least 3–4 times higher than in the general population) (12–20). Nonetheless, few studies have investigated the consequences of the COVID-19 pandemic on the health status of people experiencing homelessness, either in terms of morbidity or mortality (9, 21–24). Most of these studies have limited their design to a cross-sectional approach, with the results mainly related to the first wave of the pandemic (from March to August 2020). A recent cross-sectional study tracked changes in mortality among people experiencing homelessness from March, 2020 to March, 2021 (25). This study showed a dramatic increase in mortality during the first wave, primarily due to non-related COVID-19 causes; it may reflect the direct impact of the COVID-19 pandemic on health and social services with temporal closures and decreased shelter capacity, or on food distribution services. However, it remains unclear whether the succession of testing, screening and containment strategies toward people who are homeless during the different waves had an impact on the rate of hospitalization, i.e., on the severity of illness among people experiencing homelessness. The present study was designed to address this issue.

The objective was threefold: first, to describe COVID-19 morbidity and mortality in a large-scale cohort of individuals experiencing homelessness between June 2020 and March 2021 (the research data collected were linked to national administrative databases); Second, to compare pre-existing morbidities, symptoms and hospitalizations between people affected by SARS-CoV-2 virus and a propensity score matched sample of non-infected people in order to estimate the average marginal effect of a positive COVID-19 diagnosis; and finally, to identify factors associated with hospital admission (i.e., severe illness).

## 2. Materials and methods

### 2.1. Study design and participants

In this prospective observational cohort study, we enrolled all consecutive persons living in precarious housing conditions and accepting the proposal of a diagnosis of COVID-19 infection by serological testing between June 5, 2020 to March 31, 2021, at 48 different sites in Marseille, France. These homeless settings were identified in partnership with all the outreach teams from public health and social services and community partners working in the city, and included streets, slums, squats, emergency and transitional shelters, and drop-in centres. The living accommodations were selected based on the European Typology of Homelessness and Housing exclusion (ETHOS) classification (26). ETHOS classifies people who are homeless according to their living or housing situation, divided into 13 operational categories. We considered the following ones to address the difficulties of living conditions: – Ethos 1: People living rough; − Ethos 2: People in emergency accommodation; − Ethos 3: people living in hostels or transitional accommodation; and – Ethos 8: people in squats or shanty towns. Additional inclusion criteria were being 18-year-old at the time of the testing (16). This study covered approximately 40% of the eligible population experiencing homelessness in Marseille at a daily census point.

All participants were offered rapid serological testing. In presence of any SARS-CoV-2 suspected symptoms, a point-of-care PCR assay was performed. Only participants who received SARS-CoV-2 testing were included in the analysis.

### 2.2. Procedures

Homeless facilities register from each outreach team and users’ register from each enrolled facility during a time period were both considered in the study. We obtained sociodemographic, environmental, and clinical outcome data using a case report form (CRF). Data on living movements during the study period were also collected and obtained by direct communication with the participants. Regarding medical data, doctor or nurses conducted a medical interview with the person to determine the presence of certain morbidities (using a modified cumulative illness rating scale CIRS) (27).

The definition of the SARS-CoV-2 status was established before the start of the data collection. Broadly, positive cases consisted of participants with a positive test either based on a rapid serological test or a POC PCR test over the study period. The definition of COVID-19 symptoms also evolved at an international level as the pandemic progressed (28). When a new symptom was identified in the national and international guidelines, this was reported to all recruiting health care professionals. SARS-CoV-2 status and clinical outcomes were followed up to March 31, 2021.

A set of screening tools was adapted to the high mobility of the target population: validated serological tests manufactured by the French company Biosynex (Biosynex COVID-19 BSS) that detect *via* finger pricking the presence of immunoglobulins M (IgM) and G (IgG) within 10 min. In the validation test phase, the serological assay showed sensitivity of 91.8% (95%CI: 83.8–96.6%), specificity of 99.2% (95%CI: 97.7–99.8%) for IgM antibodies (based on 456 samples) and 100% (95%CI: 96.1–100%) and 99.5% (95%CI: 98.1–99.9%), respectively, for IgG (based on 446 samples).^1^ Sensitivity of this point-of-care serological test was assessed by an observational study and a comparative one which both reported similar specifications than the manufacturer (29–31). In addition, participants presenting with clinical signs of SARS-CoV-2 were screened using a rapid point-of-care (POC) RT-PCR test from a pharyngeal swab, which requires no laboratory handling or sample pre-processing (Biosynex vitaPCR®). The 20-min response time of the POC RT-PCR tests allows for real-time decisions and rapid dispatching of COVID-19 infected persons to specific locations for the isolation of infected people, including emergency departments and hospitals.

Rapid serological tests were performed by the mobile research team at three points: (1) at the inclusion in the cohort (June 5, 2020 to August 5, 2020), (2) 3 months later (September 11, 2020 to November 30, 2020), and (3) 6 months later (December 18, 2020 to March 31, 2021). In case of SARS-CoV-2 symptoms, the participant was treated by the mobile team according to the procedures put in place since the beginning of the epidemic and following the recommendations of the French health authorities (32, 33).

### 2.3. Outcomes

For all participants, we collected sociodemographic data, life style data, testing history, comorbidities, initial symptoms of COVID-19 infection, and COVID-19 status 3 months and 6 months after the initial interview to determine the progression of the disease among the cohort. We investigated comorbidities based on their prevalence in the cohort and their clinical relevance to the SARS-CoV-2 research field (34, 35) (i.e., obesity, diabetes, chronic respiratory pathology, cardiovascular pathology, chronic renal failure with dialysis, cancer and psychiatric or addictive comorbidities).

Our main outcomes were death, COVID-19-related hospitalizations, and all-causes hospitalizations. The mortality rate, as well as the causes of death, were first documented directly by the mobile outreach teams. Any proof of life or death and causes of death were retrieved through a thorough retrospective investigation done among each outreach team, friends, social and medical institutions, and administrative databases. For the latter, a main source was used: the French national database of deceased person that registered all deaths occurring in France (whatever the nationality of the deceased person) as well as those occurring abroad but involving French citizens. However, to use this database, the identity (first and last name) of the deceased had to be known as well as the information transmitted by the local authority where the death occurred. In cases where this identity was not known, we investigated the vital status of cohort participants in additional sources: hospital databases and database of a non-governmental organizations (NGOs) « Dead of street people ». The field coordinator was in charge of collecting data from administrative databases. We collected any hospital admission, whether or not related to a COVID-19 diagnosis, that occurred during the study period. We consulted administrative dataset from the major public hospitals and community care facilities for people experiencing homelessness in the city to inform the clinical data.

The morbidity from SARS-CoV-2 was defined according to the severity of the disease. We distinguished three levels of disease: (i) severe disease, if any hospitalization for COVID-19 occurred during the study period; (ii) moderate disease, defined based on the COVID-19-related clinical data as collected during the interviews. Following the guidelines of the French High Committee of Public Health (Haut Comité de la Santé Publique – HCSP) (36), we reported a moderate disease if any of the following symptoms were present: fever, cough, dyspnoea, headache, anosmia, rhinitis, fatigue, diarrhoea, joint pain, odynophagia, chills, mottling, skin rash and conjunctivitis; (iii) asymptomatic infections were defined by positive serological tests without COVID-19-related symptoms reported by the participants alongside the study period.

### 2.4. Statistical analysis

Categorical variables were presented as frequencies and percentages, continuous variables as means and standard deviations. The proportion of missing data was specified when greater than 5%. Categorical variables were compared by Chi-2 test or Fisher’s exact test, and continuous variables by either Student’s *t*-test or Mann–Whitney *U*-test. We compared the proportion of SARS-CoV-2-positive cases with demographic characteristics, living conditions, health characteristics and comorbidities. The proportions of the main endpoints (death and hospitalization) were estimated with its 95%CI in each study group (with or without COVID-19 infection). We used propensity score matching to estimate the average marginal effect of a positive COVID-19 diagnosis on morbidity and mortality, by controlling for confounding factors that might influence the primary outcomes. We attempted 1:3 nearest neighbor propensity score matching with a propensity score estimated using generalized linear model (GLM) regression of the COVID-19 status on the covariates. Covariates were defined by sex, age, nationality (French or other), time since precarious living conditions (1 year or less vs. more than 1 year) and month at inclusion (measurement during the first testing period, month 1–3). After matching, all standardized mean differences for the covariates were below 0.06, except for month at inclusion (0.10) and values of variance ratios were close to one, indicating adequate balance. We described and compared the clinical symptoms reported by COVID-19-negative or –positive participants using Chi-2 test or Fisher’s exact test. We carried out a false discovery rate correction of the *p-*values for symptoms to account for the increased risk of error linked to the multiplicity of tests carried out (37). Logistic regression analyses were then performed to confirm the association between COVID-19 status and comorbidities or hospitalization, after adjusting for the following main confounding factors: age, nationality, living conditions, and length of homelessness. In the COVID-19 infected group, a binary logistic regression model was run to determine the factors associated with hospital admission (i.e., severe illness), using the same adjustment variables. All tests were two-sided, and *p-*values less than 0.05 were considered statistically significant. Statistical analysis was performed using RStudio version 4.1.3.

### 2.5. Ethics statement

This study was approved by the Committee for Personal Protection (CPP-Ile-de-France VI Groupe Hospitalier Pitié-Salpetrière) on May, 2020 (number 44–20). Written informed consent was obtained from all participants. Participants who objected to the use of their data or withdrew their consent during the study were excluded from the analysis.

## 3. Results

### 3.1. Cohort characteristics

A total of 1,332 participants who consented for and who received at least one serological test were included from June 5, 2020 to November 30, 2020. Supplementary Figure S1 presents the study cohort flow chart. Of these, 933 (70.1%) were men, 262 (19.7%) had French nationality and the mean age was 40.1 years (SD 14.2) (Table 1). In total, 452 (34.1%) were living in emergency shelters, 525 (39.6%) in transitional accommodations, 174 (13.1%) in squats/slums and 176 (13.3%) on the street. Only 28% declared to be homeless for less than 1 year.

**Table 1:** Comparison of the sociodemographic characteristics of the study population, between COVID-19 positive participants and negative participants (*N* = 1,332).

Sociodemographic characteristics	Total *N* = 1,332	Non-COVID-19 participants *N* = 1,120	COVID-19 participants *N* = 192	*P*-value
		*n* (%) or mean [SD]	*n* (%) or mean [SD]	
Gender				
Men	933 (70.1)	781 (69.7)	138 (71.9)	0.521
Women	399 (29.9)	339 (30.3)	54 (28.1)	
Age, year	40.1 [14.2]	39.4 [13.8]	43.1 [14.3]	**<0.001**
French Nationality^a^ (% yes)	262 (19.7)	223 (19.9)	33 (17.2)	0.362
Country of Birth^$,£^				**<0.001**
France	245 (19.6)	209 (19.9)	31 (17.0)	
European union	208 (16.6)	183 (17.4)	19 (10.4)	
Outside European union	226 (18.1)	207 (19.7)	18 (9.9)	
Africa	286 (22.9)	229 (21.8)	56 (30.8)	
Other	285 (22.8)	223 (21.2)	58 (31.9)	
Education attainment				
No educational achievement	631 (51.7)	548 (53.0)	79 (44.5)	0.057
Lower secondary	336 (27.5)	280 (27.1)	51 (28.6)	
Upper secondary or vocational	254 (20.8)	205 (19.8)	48 (26.9)	
Civil status				
Living with family	436 (34.5)	388 (36.5)	43 (23.5)	**0.004**
Isolated adult	694 (55.0)	563 (53.0)	119 (65.0)	
Isolated parent	132 (10.5)	111 (10.5)	21 (11.5)	
Having work-related resources (% yes)	101 (10.4)	94 (11.7)	6 (3.8)	**0.007**
Total length of homelessness				
<1 year	340 (27.9)	292 (28.5)	43 (23.8)	0.141
1–5 years	463 (38.0)	379 (37.0)	81 (44.7)	
>5 years	415 (34.1)	353 (34.5)	57 (31.5)	
Typology ETHOS* at baseline ^$^				
Street	176 (13.3)	145 (13.0)	24 (12.5)	**<0.001**
Emergency shelters	452 (34.1)	351 (31.4)	97 (50.5)	
Transitional shelters	174 (13.1)	150 (13.4)	24 (12.5)	
Squats, slums	525 (39.6)	471 (42.2)	47 (24.5)	

^a^The proportion of ‘No French nationality’ can be deduced; ^$^missing data were less than 3% and were not reported. ^£^“European Union” countries: Belgium, Bulgaria, Germany, Hungary, Italy, Poland, Portugal, Romania, Czech Republic, Slovakia, and Spain. “Outside European Union” countries: Albania, Armenia, Bosnia, Croatia, Moldavia, Montenegro, Serbia, Russia including Chechenia, and Ukraine. *ETHOS: the European typology for homelessness and housing exclusion. SD: standard deviation. Values in bold indicate a statistically significant difference.

### 3.2. Status toward COVID-19 infection

Among our cohort of individuals experiencing homelessness, 192 (14.4%, *N* = 1,332) were tested positive for COVID-19 during the study period (either during the different periods of rapid screening by TROD serological test or point-of-care PCR performed in the presence of symptoms, or during a hospitalization). Table 1 compares the sociodemographic characteristics of participants who had COVID-19 with healthy participants. COVID-19 positive participants differed in terms of age, with positive participants being older than their healthy counterparts (mean age 43.1 [14.3] vs. 39.4 [13.8] respectively, *p* < 0.001); a lower proportion of COVID-19-infected participants reporting work-related resources (3.8% vs. 11.7%, *p* = 0.007); a higher proportion of positive participants were born outside Europe (72.6% vs. 62.7%; *p* < 0.001). Finally, an important finding was the difference in the prevalence of COVID-19 in the population according to the type of accommodation: more than one fifth of the cohort living in emergency accommodations (either emergency shelters or hostels) were diagnosed with SARS-CoV-2 virus, whereas it was less than 15% in the other living places (*p* < 0.001).

### 3.3. Outcomes

The 192 COVID-19-positive participants experiencing homelessness were matched to 553 controls (i.e., participants who were homeless and non-COVID-19 infected). The characteristics of the matched cohort (N = 745) were similar to those in the unmatched cohort (N = 1,332), except that the proportion of people with resources from work was no longer significantly different between COVID-19-positive participants and matched controls (Supplementary Table S1).

#### 3.3.1. Mortality and morbidity associated with COVID-19

Three deaths occurred in the cohort: 2 (1%) among the COVID-19 positive participants (*N* = 192) and 1 (0.2%) among the COVID-19 negative participants (*N* = 553). While the cause of death was identified in those who tested positive (i.e., coma at hospital and liver disease), the cause of death was unknown for the negative participant.

As expected, participants with a positive COVID-19 status reported symptoms significantly more often than matched participants without infection (Table 2). For example, among those who tested positive, 20% reported fever or fatigue during the acute phase of infection and around one-quarter reported headache or cough, compared with less than 5% of their non-COVID-19 counterparts. Other symptoms (i.e., diarrhoea, dyspnoea, joint pain or skin rash) although less frequently reported by participants were found in higher proportion among participants infected compared to COVID-19-negative participants.

**Table 2:** Symptoms recorded in the last 3 months prior to serological or virological testing among participants reporting symptoms associated with COVID-19 disease.

	Non-COVID-19 matched participants *N* = 553	COVID-19 participants *N* = 192	*P*-value
Symptoms	*n* (%)	*n* (%)	
Fever	21 (5.0)	36 (19.7)	<0.001*
Cough	22 (5.2)	25 (13.7)	<0.001*
Diarrhoea	6 (1.4)	10 (5.5)	0.004*
Headache	22 (5.2)	28 (15.3)	<0.001*
Anosmia	8 (1.9)	20 (10.9)	<0.001*
Rhinitis	21 (5.0)	20 (10.9)	0.007*
Fatigue	17 (4.0)	36 (19.7)	<0.001*
Dyspnea	15 (1.4)	15 (7.5)	<0.001*
Joint pain	5 (1.2)	20 (10.9)	<0.001*
Odynophagia	5 (1.2)	20 (10.9)	<0.001*
Chills	14 (3.3)	14 (7.7)	0.020
Mottling	1 (0.0)	1 (0.5)	0.302
Skin rash	0 (0.0)	2 (1.1)	0.091
Conjunctivitis	5 (1.2)	6 (3.3)	0.077
No. of missing data (%)	129 (23.3)	10 (5.2)	
Hospital admission (% yes)^$^	66 (11.9)	73 (38.0)	**<0.001**

Chi-2 test or Fisher’s exact test were applied depending on the number of the observations. All tests are two-sided. *P-value adjusted for multiple tests using FDR method. ^$^Hospitalizations were taken into account whether or not they were related to the COVID-19 infection. Values in bold indicate a statistically significant difference.

**Figure 1:**
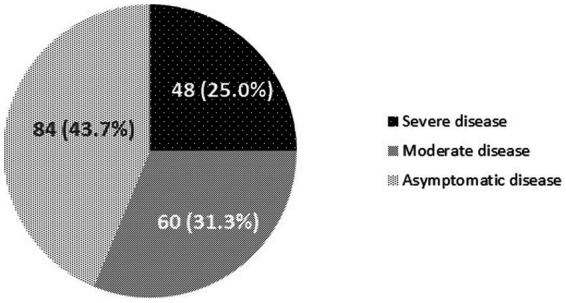
Number of cases and proportions of those with severe, moderate and asymptomatic COVID-19 infections, over the study period (May 2020 to March 2021). Severe disease: asymptomatic or symptomatic disease requiring hospital care, Moderate disease: presence of symptoms not requiring hospital care, and Asymptomatic disease: asymptomatic and benign infection not requiring hospital care.

Of the 192 COVID-19-infected individuals, 48 (25.0%) positive participants were hospitalized due to severity of illness, leading to estimate a cumulative incidence rate of hospitalization related to COVID-19 of 3,600 hospitalizations per 100,000 persons experiencing homelessness per year in France. This rate was considerably higher than in the general population in Marseille or at a national level during the same period. Data from the French National Public Health Agency show that the national incidence rate COVID-19 hospitalisation was 781.8 per 100,000 French adults between March 2020 and March 2021 (38). Symptoms not requiring hospital care were reported by 60 (31.3%) COVID-19-infected participants, with the remainder (43.7%) having asymptomatic, benign infection (Figure 1).

Taking all hospitalizations into account, whatever the cause (COVID-19 and non-COVID-19 related), 139 (18.7%) people were hospitalized for all causes (Table 2). The rate of hospitalized participants was 4 times higher in the COVID-19-infected group than in the non-infected group [*p* < 0.001; odds ratio adjusted (aOR) for age, nationality, living conditions and length of homelessness: aOR 4.21; 95%CI 2.84–6.25] (results not shown).

#### 3.3.2. Pre-existing comorbidities as risk factors for hospitalization

The proportion of participants with pre-existing comorbidities was higher among those who have COVID-19 (both severe and non-severe cases) than among matched control participants, with the most important difference for cardiovascular pathology and musculoskeletal disorders (Table 3). Respectively, less than 12, 8 and 4% of matched participants had lung problems, obesity, and cancer or kidney disease, with no significant differences between groups. Consumption of tobacco or alcohol was significantly lower in the participants with COVID-19 compared with their matched counterparts (*P* ≤ 0.002). The use of other substances (such as cannabis, cocaine or opioid agonists) was not significantly different between the two matched groups (*p* = 0.113). Compared with the matched non-COVID-19 infected participants, COVID-19-positive participants were significantly more likely to report cardiovascular pathology (aOR 1.68; 1.02–2.77), musculoskeletal disorders (aOR 2.80; 1.44–5.43), or at least 3 pre-existing comorbidities (aOR 1.85; 1.16–2.94).

**Table 3:** Univariate and multivariate logistic regression analysis of the factors associated with COVID-19 disease or severe disease, i.e., requiring hospital care, referent to, respectively, non-COVID-19 disease or non-severe disease, i.e., asymptomatic and symptomatic patients not requiring hospital admission.

	Non-COVID-19 matched participants *N* = 553	Non-severe COVID-19 participants *N* = 144	Severe COVID-19 participants *N* = 48	Non-COVID-19 vs. COVID-19 participants		Non-severe vs. Severe COVID-19 participants	
	*n* (%) or mean [SD]	*n* (%) or mean [SD]	*n* (%) or mean [SD]	*P*-value univariate*	aOR (95%CI)	*P*-value univariate*	aOR (95%CI)
Addiction behavior							
Tobacco consumption (% yes)	314 (56.8)	61 (42.4)	21 (43.8)	**0.001**	**0.58 (0.42–0.81)**	0.866	0.82 (0.39–1.71)^§^
Alcohol consumption (% yes)	154 (29.7)	22 (15.8)	11 (26.2)	**0.002**	**0.52 (0.3–0.79)**	0.214	1.79 (0.75–4.29)^§^
Other substances consumption (% yes)^μ^	114 (20.6)	22 (15.3)	8 (16.7)	0.113	0.71 (0.45–1.12)	0.819	1.29 (0.51–3.27)
Cannabis (% yes)	93 (16.8)	18 (12.5)	6 (12.5)				
Cocaine (% yes)	35 (6.3)	6 (4.2)	2 (4.2)				
Opioid agonist (% yes)	21 (3.8)	3 (2.1)	1 (2.1)				
Polysubstance use^$^	173 (33.6)	31 (23.8)	14 (31.1)	0.053	0.67 (0.44–1.01)	0.337	1.42 (0.62–3.67)
Type of pre-existing comorbidities							
Psychiatric and addiction comorbidities (% yes)	94 (17.7)	26 (18.3)	15 (31.2)	0.236	1.32 (0.85–2.03)	0.060	2.06 (0.93–4.45)^§^
Cardiovascular Pathology (% yes)	68 (14.6)	25 (20.7)	11 (26.2)	**0.027**	**1.68 (1.02–2.77)**	0.457	1.02 (0.42–2.50)
Musculoskeletal disorders (% yes)	22 (4.4)	10 (7.9)	9 (20.5)	**0.002**	**2.80 (1.44–5.43)**	**0.023**	2.58 (0.92–7.08)
Diabetes (% yes)	42 (8.6)	13 (10.6)	9 (20.5)	0.078	1.52 (0.85–2.70)	0.109	1.94 (0.71–5.30)
Chronic Respiratory Pathology (% yes)	44 (9.4)	15 (12.7)	3 (7.3)	0.479	1.29 (0.71–2.36)	0.567	NA
Obesity (% yes)	29 (6.0)	8 (6.6)	4 (9.8)	0.515	1.27 (0.63–2.59)	0.501	NA
Cancer (% yes)	11 (2.3)	3 (2.4)	4 (9.8)	0.185	2.04 (0.76–5.50)	0.066	NA
Chronic renal failure (% yes)	10 (2.1)	4 (3.4)	2 (5.0)	0.257	1.58 (0.56–4.47)	0.645	NA
> = 3 risk factors of severe COVID-19 infection (% yes)^£^	66 (12.4)	24 (16.9)	15 (31.2)	**0.007**	**1.85 (1.16–2.94)**	**0.040**	1.70 (0.76–3.79)^§^

*Fisher’s exact test for chronic respiratory pathology, obesity, cancer and chronic renal failure variables, χ2 test for other variables. All tests are two-sided. aOR: Adjusted Odds Ratios. ORs were adjusted (by regression) for age, nationality, living conditions, and length of homelessness. ^μ^most reported used drugs, i.e., cannabis, cocaine (coke, crack) and opioid agonists. ^$^Polysubstance use was defined as use of more than one substance (tobacco, alcohol, other substances). ^§^Age was the only variable significantly associated with being severely infected. ^£^Potential risk factors for severe COVID-19 disease, including cancer, obesity, cardiac or pulmonary disease and severe renal insufficiency. Values in bold indicate a statistically significant difference.

Table 3 also provides the adjusted odds ratio of the factors associated with severe COVID-19 disease, that is requiring hospital care, referent to non-severe disease. In the 192 individuals who had been infected (i.e., *N* = 48 for severe COVID-19 cases and *N* = 144 for non-severe COVID-19 cases), only older age (aOR 1.04; 1.02–1.06; *p* = 0.003) was associated with increased risk of hospitalization.

## 4. Discussion

This study is an attempt to quantify the mortality as well as the morbidity of COVID-19 among the people who were homeless throughout the three first waves of the pandemic in France.

Among the types of accommodations for people experiencing homelessness, emergency shelters represented the greatest risk of exposure to COVID-19. Seroprevalence among people living in emergency shelters, estimated in our cohort at 22%, was extremely higher than in the national seroprevalence survey on “Epidemiology and Living Conditions” (EPICOV), conducted over the same period (39). In this French study based on 12,000 individuals tested between May and June 2020, a positivity rate of 4.5% was reported nationwide, with a positivity rate of 3.6% in the city of Marseille. Similar sociodemographic characteristics of those most at risk of the epidemic were found in our study and in the published literature, namely, being born outside Europe or living in crowded accommodations (15, 18, 23, 40, 41).

While mortality among COVID-19 infected participant remained very low and no reported deaths were related to SARS-CoV-2 virus during the follow-up period, our finding of a higher hospitalization rate among COVID-19 positive participants is consistent with the major published literature on COVID-19, as reported in a scoping review of Corey et colleagues (42). With 25% of participants who developed COVID being hospitalized, this rate is considerably higher than in the general population in Marseille or at a national level during the same period. More precisely, among French people in the same age range (i.e., average age 40–45 years) 7.4% were infected in the year 2020; of these, 3.4% were hospitalized (43). Similar results were found in the literature (23, 44). In the latter, a serological study conducted in France among shelter residents during the first wave, the hospitalization rate was 23.6%.

Predisposing conditions such as addictions, psychiatric disorders, comorbidities, as well as exposure to communicable diseases in the population experiencing homelessness have been shown to be significantly associated with higher rates of hospitalization compared to their housed counterparts (45). In the context of the COVID-19 pandemic, this interdependence between homelessness and hospitalization has been further strengthened with a greater prevalence of COVID-19 in the population experiencing homelessness.

In our study, a high hospitalization rate was associated with an older age in COVID-infected participants and we found no evidence of higher risk due to pre-existing comorbidities or living conditions (46). Our findings are consistent with the results of two epidemiological studies conducted in France, the EpiCoV survey and SAPRIS survey “Health, Perception, Practices, Relations and Social Inequalities in the General Population during the COVID-19 crisis,” which have also shown a strong relationship between being COVID-positive and age (41, 47).

In the present study, we found a high proportion of asymptomatic adults among the positive-COVID-19 participants who were homeless (42.7%). A recent meta-analysis reported a rate of 65% in the same population (48). These findings point to the need for broad and regular screening programs among the population experiencing homelessness to prevent clusters of infection in crowded or inadequate accommodations. Then, we found that COVID-19 infection was associated with a range of pre-existing comorbidities; the strongest associations were observed for psychiatric/addiction disorders and cardiovascular pathology. Among those with severe COVID-19 infection, musculoskeletal disorders were also present in a high proportion (>20%). Finally, a high prevalence of pre-existing comorbidities was associated with an increased risk of developing severe COVID-19 infection; this relationship was highly significant between non-infected individuals and those with COVID-19 disease requiring hospital care; whereas a simple trend was observed among infected individuals not requiring hospital care. Similar trends were reported in the literature in both general and vulnerable populations (8, 21, 23, 49).

Different interventions to mitigate the spread of COVID-19 (and other respiratory viruses) among people who are homeless have been proposed since the first wave of the pandemic. Some of them focus on providing essential needs (such as foods, hygiene) during crisis and insist on the critical role that community-based organizations, field workers have in adapting support services to respond to the needs of socially excluded populations during public health crisis. One of the main strategies would be to better manage mental and physical health issues, as well as substance use disorders, by offering rapid in-person support services. Two forms of services could be developed: either by offering a virtual format of care, implemented as needed and where computer/phone access and skills are available, or by expanding targeted outreach teams in more dedicated way, around connections to mental health and substance abuse treatment services (50). Finally, provision of housing solutions is key. Although housing teams faced specific issues during the pandemic, studies have showed that such housing solution overcame the challenges brought by the COVID-19 infection among the vulnerable population. These challenges referred mainly to rapid coordination of efforts during outbreak-related shelter shutdowns, strong partnerships between community care facilities for people experiencing homelessness, local non-governmental organizations with local health department. In addition, the State provided a constant source of guidance to first isolation of infected people and then housing solutions (51–53). Nevertheless, the most critical point is targeting effective prevention strategies including COVID-19 vaccination efforts among people experiencing homelessness. In France, COVID-19 vaccination strategy targeting people who are homeless was implemented in May 2021, including management of vaccination centers for marginalized population, sensitization, mobile vaccination teams, and physical accompaniment to vaccination centers (54).

The main study strengths include the prospective design of the study and the systematic inclusion of the population, with the enrolment of approximately 40% of the population experiencing homelessness in Marseille. The research also led to the development of an outreach intervention to better address the complex needs of persons who are homeless. The main factors behind the success of this innovative intervention can be described as follows: (i) an outreach model, with fast interventions by field workers directly at locations frequented by people experiencing homelessness; (ii) a large amount of field professionals (social workers and health professionals) monitoring their users and soliciting our team in case of clinical suspicion of COVID-19; (iii) the involvement of peers as community mediators to approach communities with respect and empower teams; (iv) a set of adapted screening tools (serological rapid tests and a mobile PCR machine for free virological testing, with a response within 20 min), enabling rapid orientation of persons with positive results toward specific accommodation reserved for the isolation of people who were homeless with COVID-19; (v) clinical follow-up of positive and negative cases (with serological and/or virological retesting if necessary); (vi) individualized counselling and/or the implementation of appropriate accommodation measures (with the support of local authorities), such as the provision of free single rooms for the most vulnerable persons, making efforts to alleviate the density at shelters by making use of hostels, supplying masks, providing advices on ventilation and interventions to connect slums to water and soap distribution, etc.; (vii) coordination with Government and the local structures, such as medical analysis laboratories, and the Governmental instances responsible for contact-tracing and monitoring contamination-clusters, has also been a key factor in this model. The main limitation of this study was the lack of systematic follow-up data from the homeless cohort during the different waves and lockdowns due to their movements from one place of accommodation to another, or from one place of isolation for COVID-19 infection to a squat or shelter. A first consequence was the patchy data on people’s vital status and the possible underestimation of the burden of COVID-19 in this population. A second consequence was the presence of non-monotone missing data (i.e., a missing variable for a particular individual does not imply that all subsequent variables are missing for that individual) inherent in intervention research, especially in health crisis situations. The field teams sometimes had to deal with the urgency of COVID-19 or cluster situations and neglected to fill in the questionnaires and patient files. The mobility of the cohort, even if limited during this period, may have been accompanied by an underestimation of the hospitalization number. Data on use of mechanical ventilation or long COVID-19 were missing from most patients at the time of analysis. Similarly, the number of chronic diseases might be underdiagnosed. Another limitation was the difficulty of certifying identities within national registers using self-reported foreign surnames, some of which were either difficult to spell or very common in the communities.

## 5. Conclusion

The results of this study provide evidence that the population experiencing homelessness faces higher risks of infection and hospitalisation for COVID-19 than the general population. Despite the efforts of public authorities, the health inequities experienced by people experiencing homelessness remained major. The study also highlights that more intensive and appropriate integrated care and earlier re-housing are needed to mitigate the spread of COVID-19 among people experiencing homelessness.

## Data availability statement

The raw data supporting the conclusions of this article will be made available by the authors, without undue reservation.

## Ethics statement

The studies involving humans were approved by Committee for Personal Protection (CPP-Ile-de-France VI Groupe Hospitalier Pitié-Salpetrière) on May, 2020 (number 44-20). The studies were conducted in accordance with the local legislation and institutional requirements. The participants provided their written informed consent to participate in this study.

## Author contributions

SL and IH: first draft of the manuscript. SL, IH, EmM, ElM, AT, and PA: subsequent drafts. TB and MM: data collection. SL and IH: data analysis. AT, EmM, and SL: study design. All authors made substantial contribution to the interpretation of data and agreed with the content of the final manuscript.

## Footnotes

^1^ www.biosynex.com
