# Supplementary material for: Morbidity and mortality in a prospective cohort of people who were homeless during the COVID-19 pandemic
Source: Front Public Health. 2023 Sep 14;11:1233020. doi: 10.3389/fpubh.2023.1233020 (PMC10536263; doi:10.3389/fpubh.2023.1233020)
Supplement: Supplementary file 1 [file Table_1.docx]

***Supplementary Material***

**Morbidity and mortality in a prospective cohort of people who were homeless during the COVID-19 pandemic**

Sandrine LOUBIERE*, Ikrame HAFRAD, Elisabetta MONFARDINI, Marine MOSNIER, Thomas BOSETTI, Pascal AUQUIER, Emilie MOSNIER, Aurélie TINLAND

***Correspondence:** Sandrine Loubiere: [sandrine.loubiere@univ-amu.fr](mailto:sandrine.loubiere@univ-amu.fr)

**1 Supplementary Table S1**

**Table S1: Comparison of the sociodemographic characteristics between matched population (COVID-19 positive participants and negative participants (N=745).**

| *Sociodemographic characteristics* | Non-COVID-19  matched  participants  N=553 | COVID-19  Participants  N=192 | *P-value* |
| --- | --- | --- | --- |
|  | **n (%) or mean**  **[SD]** | **n (%) or mean [SD]** |  |
| Gender, Men | 395 (71.4) | 138 (71.9) | 0.906 |
| Age, year | 40.7 [13.8] | 43.1 [14.3] | 0.046 |
| French Nationality ^a^ (% yes) | 112 (20.3) | 33 (17.2) | 0.355 |
| Country of Birth^$,£^ |  |  | **<0.001** |
| France | 106 (19.4) | 31 (17.0) |  |
| European union | 104 (19.3) | 19 (10.4) |  |
| Outside European union | 106 (19.6) | 18 (9.9) |  |
| Africa | 126 (23.3) | 56 (30.8) |  |
| Other | 98 (18.1) | 58 (31.9) |  |
| Education attainment |  |  |  |
| No educational achievement | 268 (51.4) | 79 (44.3) | 0.094 |
| Lower secondary | 152 (29.1) | 51 (28.7) |  |
| Upper secondary or vocational | 102 (19.5) | 48 (27.0) |  |
| Civil status |  |  |  |
| Living with family | 190 (34.9) | 43 (23.5) | **0.016** |
| Isolated adult | 298 (54.8) | 119 (65.0) |  |
| Isolated parent | 56 (10.3) | 21 (11.5) |  |
| Having work-related resources (% yes) | 37 (9.3) | 6 (3.8) | 0.058 |
| Total length of homelessness |  |  |  |
| < 1 year | 134 (25.7) | 43 (23.8) | 0.282 |
| 1 to 5 years | 199 (38.1) | 81 (44.7) |  |
| >5 years | 189 (36.2) | 57 (31.5) |  |
| Typology ETHOS* at baseline ^$^ |  |  |  |
| Street | 80 (14.5) | 24 (12.5) | **0.002** |
| Emergency shelters | 201 (36.5) | 97 (50.5) |  |
| Transitional shelters | 61 (11.1) | 24 (12.5) |  |
| Squats, slums | 170 (37.9) | 47 (24.5) |  |

a: the proportion of 'No French nationality' can be deduced; $: missing data were less than 3% and were not reported. £: “European Union” countries: Belgium, Bulgaria, Germany, Hungary, Italy, Poland, Portugal, Romania, Czech Republic, Slovakia, and Spain. “Outside European Union” countries: Albania, Armenia, Bosnia, Croatia, Moldavia, Montenegro, Serbia, Russia including Chechenia, and Ukraine. *ETHOS: the European typology for homelessness and housing exclusion. SD: standard deviation.

**2 Supplementary Figure S1**

**Figure S1:** **Flow chart of the SARS-CoV-2 seroprevalence study in homeless population, Marseille, France**

**Original study population**^a^ **of**

**homeless people living**

**in ETHOS 1, 2, 3 and 8 conditions**

N=4000

**Not eligible ^b^:**

- Not criteria: Family accommodations and children^c^
- Refusals to consent
- Comprehension issues
- Not reachable at the time of the study took place

30%

**Total participants with signed consent**

N=1,472

**Excluded:**

- Without a valid consent form
- Withdrawals

**Total participants enrolled**

N=1,332

**Participants**

**with 3^rd^ serology**

N=599 (45%)

**Participants**

**with 1^srt^ serology**

N=1332 (100%)

**Participants**

**with 2^nd^ serology**

N=745 (56%)

**COVID-19 -Participants**

N=1,120

**COVID-19 + Participants**

N=192

**Propensity score 1:3 matching**

**Matched COVID-19-negative Participants**

N**=**553

**Matched COVID-19-positive Participants**

N=192

a: Although comprehensive homelessness prevalence data for Marseille are still lacking, we used data from local Integrated Reception and Orientation Service (IROS–SIAO in French) for emergency and transitional accommodations, and estimations of NGOs for slums/squats and streets;

b: Reasons for not being included in the study were difficult to distinguished as most of people living in squats or streets cumulated the three main reasons (refusals, comprehension issue or unreachable at the time of the study took place);

c: estimations of NGOs at around 30% of squat inhabitants.
